# Adnexal Tumor-Related Neurobehavioral Alterations: A Rare Case Report

**DOI:** 10.1155/2020/3802940

**Published:** 2020-02-25

**Authors:** Gabriel Ferrante Abou Murad, Francisco Faria Hatanaka, Tamires de Menezes França, Maria Gabriela Baumgarten Kuster Uyeda, Manoel João Batista Castello Girão, Marair Gracio Ferreira Sartori

**Affiliations:** ^1^Paulista School of Medicine, Federal University of Sao Paulo EPM-UNIFESP, Sao Paulo, Brazil; ^2^Department of Gynaecology, Escola Paulista de Medicina, Universidade Federal de São Paulo, Sao Paulo, Brazil

## Abstract

Associations between neuropsychotic changes and oncologic disease are often described as late-onset symptoms, secondary to the instituted treatment. However, neurocognitive symptoms as a clinical manifestation of adnexal tumors are still little discussed, despite the importance of these in the gynecology practice. In this article, we present a case of adnexal mass whose first clinical manifestations were neuropsychiatric alterations. Such changes led the patient to seek our health service, and after diagnosis and treatment of the ovarian mass, the patient presented remission of the symptoms.

## 1. Introduction

Adnexal masses are inherent to the gynecology practice, in any age group. A woman has a chance from 5% to 10% of having surgery due to adnexal mass in her life (Muto, 2016). The wide variety of possible diagnoses facing an additional mass lead to many possibilities regarding evolution and symptoms. Despite the lack of formal statistical data, neurocognitive alterations are described as a possible clinical manifestation of adnexal tumors. Ovarian cancer typically presents with 3–4 months of abdominal pain or distension, which might be mistakenly attributed to irritable bowel syndrome [1, 2]. Neurological and behavioral changes are often associated with leptomeningeal carcinomatosis, and it is an uncommon and typically late complication of ovarian cancer [3]. This article presents a rare case report of a 59-year-old female patient with cognitive and behavioral disorders diagnosed during research and of a benign adnexal mass, neuropsychiatric symptoms that regressed after surgery, and exerted adnexal mass.

## 2. Case Report

**Figure 1:**
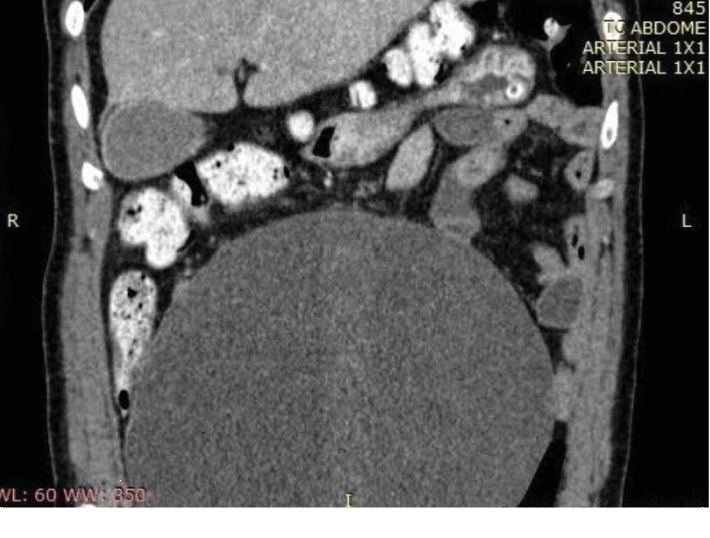
Computed tomography in coronal reconstruction.

**Figure 2:**
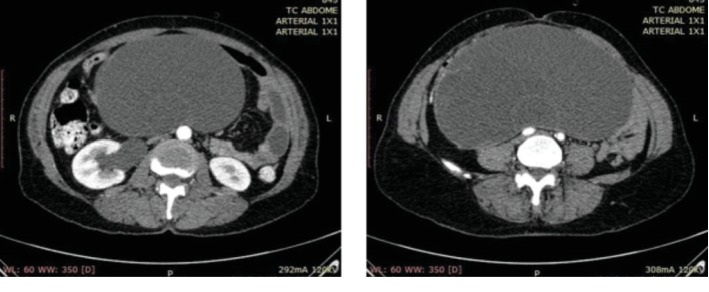
Computed tomography in axial reconstruction.

A 59-years-old woman, Caucasian, divorced, who works at home, was taken to the hospital by relatives presenting humor alterations, described as hypotimia and melancholy, inappetence, and constipation for three days. There is history of the following unspecified mental behavior for at least 10 years without diagnosis or treatment until admission to this service and diagnosis of adnexal mass. About the gynecological and obstetric history, she had menarche at fifteen and she had menopause at 42. Beginning of sexual life was at 22, having a partner in life. She had three pregnancies, two vaginal deliveries and one cesarean section. For personal history, she was unquantified long-time smoker. She denied any other health conditions. We could not gather family history due to the patient's clinical conditions upon coming to our health service. During physical examination, the patient was in regular general condition, pale, conscious, oriented in time and space, and making small contact. The abdominal examination revealed a mass of imprecise limits, hardened and nonpainful, from the pelvis to 1 cm above the umbilical scar, with no inflammatory signs. Bowel sounds were present, at a reduced frequency. The complementary investigation was made with abdominal and pelvic computed tomography, which described the abdominal mass identified at physical examination was the right annex, cystic, regular, and homogeneous. It had no contrast uptake and had large central septa (Figures 1 and 2). Measurements were 20 cm, 10 cm, and 10 cm, compressing viscera but without signs of intestinal obstruction. Other organs had no alterations. Laboratory tests showed normal electrolytes, liver tests, blood count, and clotting time. Tumor markers were also evaluated: alpha-fetoprotein; chorionic gonadotrophin (she had already done in another service); carcinoembryonic antigen; CA 19-9, all within normality; and CA 125, lightly elevated (39 U/mL, reference value up to 35).

**Figure 3:**
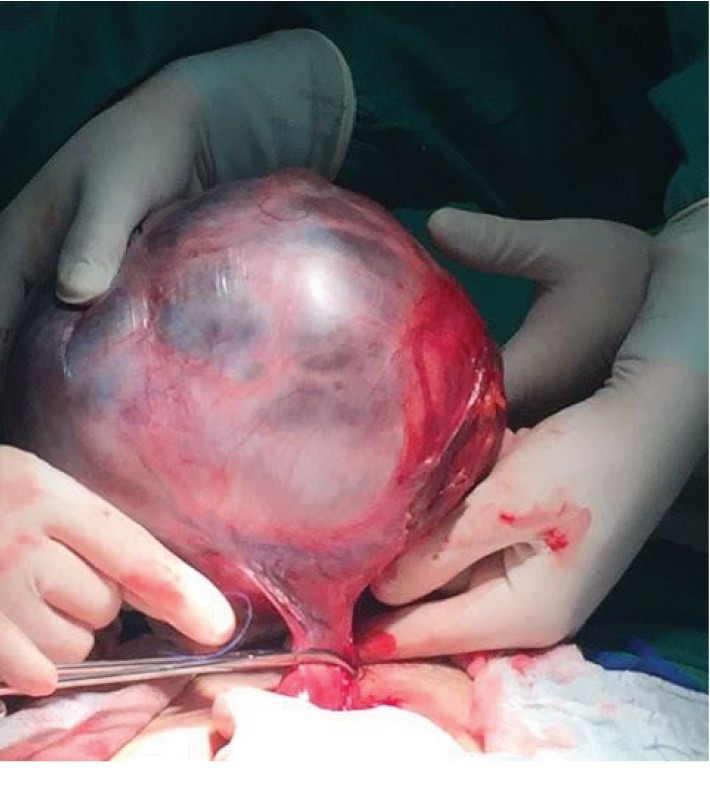
Tumor pedicle.

**Figure 4:**
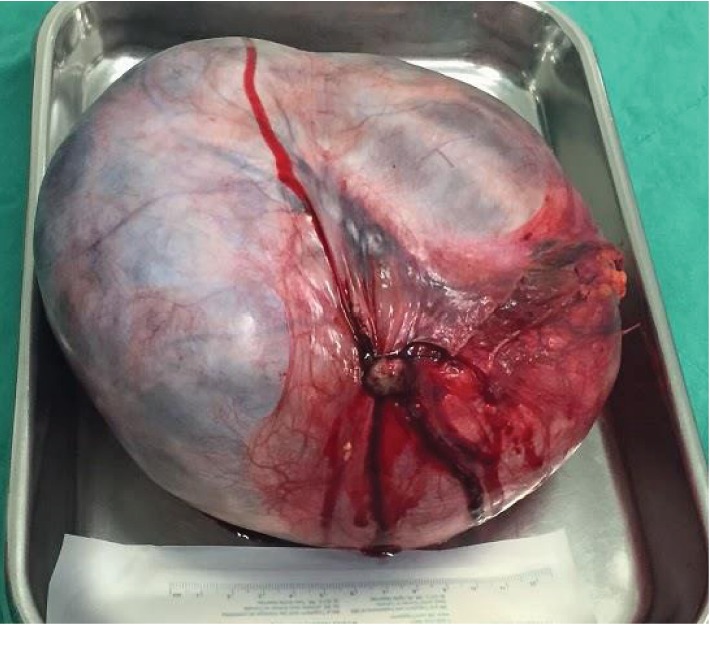
Adnexal mass after exeresis.

During her hospital stay, the patient kept neuropsychotic alterations described by our mental health team as depressed humor, slowing down of the thought process, memory impairment (difficulty to mark when facts of her life happened), and impaired criticism, with no alteration in orientation to space, to the current time, and to herself. Although there were no signs of bowel obstruction at the CT scan, it was decided to place a nasogastric tube due to the anorexia and elimination of gases and feces arrest, with debt of 1700 mL. Exploratory laparotomy was then indicated. After opening the cavity, a massive solid-cystic formation corresponding to the right ovary was observed, besides a normal-looking uterus and left ovary (Figure 3). There was absence of ascites, peritoneal implants, or adhesions. It was decided to proceed with total hysterectomy, bilateral salpingo-oophorectomy, and peritoneal lavage collection. Frozen section was not sent intraoperatively; service was not available at the hospital at that time the anatomic-pathological result showed the right adnexal area measuring 14 cm × 11 cm × 6.5 cm (Figure 4), with the diagnosis of gastrointestinal mucinous cystadenoma. About the appendix, it was evaluated and did not have any abnormality.

During the postoperative period, the patient showed contact from the first day, with significant improvement of the neuropsychiatric alterations previously described: normal course of thought, form, and content, without memory impairment, preserved criticism, and euthymic humor. This improvement was maintained during the three days of hospitalization in the postoperative period, being considered, therefore, stable.

## 3. Discussion

Despite the evolution of diagnostic methods such as ultrasonography, extensive tumor growth resulting in large ovarian masses is relatively a common finding, especially in malignant tumors, in which the rapid evolution of the disease implies advanced symptomatology in a short period [4, 5]. However, ovarian mucinous cystadenoma, a tumor in which biological behavior is described as mostly benign or borderline, can reach large dimensions. Early symptoms may appear late due to the indirect effects of the large tumor mass, with compression of the adjacent pelvic and abdominal organs and large vessels [6, 7].

About the ovarian tumors, a rare group of symptoms may be present: neurocognitive disorders, detected in this case. Cancer patients as a whole may present, during disease evolution, changes in psychic functions (such as reduced attention, lack of memory, or decline in executive functions). Neurological and behavioral changes are often associated with leptomeningeal carcinomatosis, and it is an uncommon and typically late complication of ovarian cancer [3]. In this case, we have the rare report of neuropsychiatric changes in a benign ovarian tumor, with complete resolution of symptoms after the injury is exercised. Such changes can be attributed to the direct and indirect effects of central nervous system neoplasia, neurological or psychiatric comorbidities aggravated by neoplastic disease, or treatment with chemotherapy or radiotherapy [8]. However, much of what is known about the relationship between cognitive functions and oncology has been investigated in breast cancer patients, and little is known about psychic changes in other types of cancer. The evaluation of possible neurological deficits is important in patients with ovarian tumors due to the severity of the condition and its impact on the quality of life—even greater if there are cognitive sequelae [9]. The case we bring in this article shows an exuberant neuropsychotic presentation as a clinical finding of the patient's ovarian tumor, with the abdominal mass being evaluated only later. The main articles describing these findings concerning ovarian tumors show progressive evolution of symptoms, usually with disease follow-up or associated with chemotherapy and rarely in several fields of mental function simultaneously [9]. However, this case draws attention to the early involvement of several neuropsychotic domains, as well as the acute nature of the condition, and its resolution directly related to the immediate postoperative period of laparotomy to remove the ovarian mass. It is concluded that, although the symptomatology of ovarian tumors does not classically include neurocognitive symptoms, medical teams may come across clinical conditions with this presentation and should be alert and prepared to perform a detailed psychic evaluation and systemic investigation to conduct appropriate treatment and provide greater comfort and quality of life.
